# PRIMAGE project: predictive *in silico* multiscale analytics to support childhood cancer personalised evaluation empowered by imaging biomarkers

**DOI:** 10.1186/s41747-020-00150-9

**Published:** 2020-04-03

**Authors:** Luis Martí-Bonmatí, Ángel Alberich-Bayarri, Ruth Ladenstein, Ignacio Blanquer, J. Damian Segrelles, Leonor Cerdá-Alberich, Polyxeni Gkontra, Barbara Hero, J. M. García-Aznar, Daniel Keim, Wolfgang Jentner, Karine Seymour, Ana Jiménez-Pastor, Ismael González-Valverde, Blanca Martínez de las Heras, Samira Essiaf, Dawn Walker, Michel Rochette, Marian Bubak, Jordi Mestres, Marco Viceconti, Gracia Martí-Besa, Adela Cañete, Paul Richmond, Kenneth Y. Wertheim, Tomasz Gubala, Marek Kasztelnik, Jan Meizner, Piotr Nowakowski, Salvador Gilpérez, Amelia Suárez, Mario Aznar, Giuliana Restante, Emanuele Neri

**Affiliations:** 1 Medical Imaging Department, La Fe University and Polytechnic Hospital & Biomedical Imaging Research Group (GIBI230) at La Fe University and Polytechnic Hospital and Health Research Institute, Av. Fernando Abril Martorell 106, 46026 Valencia, Spain; 2 Quantitative Imaging Biomarkers in Medicine, QUIBIM SL, Edificio Europa, Av. de Aragón, 30, Planta 12, 46021 Valencia, Spain; 3 Children’s Cancer Research Institute, Vienna, Austria; 4 Instituto de Instrumentación para Imagen Molecular (I3M), Universitat Politècnica de València (UPV), c\ Camino de Vera s/n, 46022 Valencia, Spain; 5 Biomedical Imaging Research Group (GIBI230), La Fe Health Research Institute, Av. Fernando Abril Martorell 106, Torre E, 46026 Valencia, Spain; 6 Department of Pediatrics, Faculty of Medicine and University Hospital Cologne, University of Cologne, Cologne, Germany; 7 Multiscale in Mechanical and Biological Engineering, Department of Mechanical Engineering, Universidad de Zaragoza, Zaragoza, Spain; 8 Aragón Institute of Engineering Research, Zaragoza, Spain; 9 Department of Computer Science, University of Konstanz, Konstanz, Germany; 10 Medexprim, 815 La Pyrénéenne, 31670 Labège, France; 11 Paediatric Oncology Unit, La Fe University and Polytechnic Hospital, Av. Fernando Abril Martorell 106, Torre G, 2 Floor, 46026 Valencia, Spain; 12 European Society for Paediatric Oncology, Brussels, Belgium; 13 Department of Computer Science and Insigneo Institute of In Silico Medicine, University of Sheffield, Regent Court, 211 Portobello, Sheffield, UK; 14 Simulation, Modelling and Engineering Software, Ansys Group, Montigny-le-Bretonneux, France; 15 ACC Cyfronet, AGH University of Science and Technology, Sano Centre for Computational Medicine, Nawojki 11, 30-950 Kraków, Poland; 16 Chemotargets S.L., Carrer de Baldiri Reixac, 4-8 TI05A7 Torre I, planta 5, A7, 08028 Barcelona, Spain; 17 Department of Industrial Engineering, Alma Mater Studiorum, University of Bologna, Bologna, Italy; 18 Matical Innovation, Calle de Torija, 5, 28013 Madrid, Spain; 19 Department of Translational Research, University of Pisa, Chair Radiodiagnostica 3, Pisa University Hospital, Via Roma 67, 56126 Pisa, Italy

**Keywords:** Artificial intelligence, Biomarkers (tumour), Cloud computing, Diffuse intrinsic pontine glioma, Neuroblastoma

## Abstract

PRIMAGE is one of the largest and more ambitious research projects dealing with medical imaging, artificial intelligence and cancer treatment in children. It is a 4-year European Commission-financed project that has 16 European partners in the consortium, including the European Society for Paediatric Oncology, two imaging biobanks, and three prominent European paediatric oncology units. The project is constructed as an observational *in silico* study involving high-quality anonymised datasets (imaging, clinical, molecular, and genetics) for the training and validation of machine learning and multiscale algorithms. The open cloud-based platform will offer precise clinical assistance for phenotyping (diagnosis), treatment allocation (prediction), and patient endpoints (prognosis), based on the use of imaging biomarkers, tumour growth simulation, advanced visualisation of confidence scores, and machine-learning approaches. The decision support prototype will be constructed and validated on two paediatric cancers: neuroblastoma and diffuse intrinsic pontine glioma. External validation will be performed on data recruited from independent collaborative centres. Final results will be available for the scientific community at the end of the project, and ready for translation to other malignant solid tumours.

## Key points

- An open-cloud platform for decision support in neuroblastoma and diffuse intrinsic pontine glioma is being developed.
- A decision support system guided by imaging and paediatric oncology experts under a user-centric approach will be developed.
- The platform will validate imaging biomarkers (computed tomography, magnetic resonance, positron emission tomography, ^131^I-meta-iodobenzylguanidine imaging) and integrated data.
- The system will develop diagnostic multiscale models to predict disease progression.

## Background

The digital transformation of healthcare systems has fostered innovative clinical workflows and quality improvements through value-based healthcare [1]. Nowadays, digital diagnosis tools (such as imaging, pathology, genomic analytics, wearable sensors) and patient electronic records (clinical profiling, treatment, endpoints) are key enabling factors for a new paradigm in routine clinical practice. This change is expected to promote clinical innovation models via real-world data-driven inferences revealing insights implicit in the data [2]. Real-world evidence can help answering existing questions and generating new knowledge in a more reproducible way [3].

Another key enabling factor to untap the enormous potential of *in silico* tools to assist in clinical healthcare is the current level of adoption of high-throughput screening techniques for diagnosis and disease progression monitoring. The amount of clinical, pathological, molecular and imaging data available is enormous. The possibility of integrating large volumes of highly heterogeneous data into *in silico* predictive tools has proven crucial to enhance model performance in various applicability domains [4].

Computational imaging allows the extraction of multiparametric data, leading to a new era in radiomics, characterised by high-throughput extraction, storage and analysis of a large amount of quantitative imaging features and parameters (imaging biomarkers) able to provide quantitative relevant information (virtual biopsies) for the early disease diagnosis, disease phenotyping, disease grading, targeting therapies, and evaluation of disease response to treatment [5].

The development of predictive models using computational algorithms and artificial intelligence, taking into account all types of clinical, pathology, molecular, and imaging information able to predict valid disease-related outcomes by learning from retrospective data, is a hot topic of scientific debate. The validity of these predictive models depends on the quantity, quality, and representativeness of the datasets used, being major limiting factors [6].

### Imaging biobanks and *in silico* models

Oncologic imaging represents a suitable field for the discovery and validation of new biomarkers from different imaging modalities (such as computed tomography, magnetic resonance, positron emission tomography, and ultrasound), since cancer patients are frequently monitored for staging and treatment response follow-up [7]. Many imaging biomarkers have been proposed over the last years to measure tumour anatomy, morphology, pathophysiology, metabolism, or molecular profiles in order to estimate different cancer hallmarks, such as proliferation/growth, angiogenesis, and evasion or metastasis [8]. However, very few biomarkers have so far entered routine clinical practice to guide clinical decisions [9, 10]. The majority of oncology imaging biomarkers still require external validation at different centres before they can be properly qualified as robust and reproducible.

Mathematical and computational modelling of biological processes can be used to enhance quantitative understanding of biomedical phenomena, such as cancer progression [11], potentially incorporating patient-specific data to enrich the scope of therapeutic target identification. Models can describe the growth of solid tumours using discrete or continuous representations, with or without accounting for stochasticity [12]. PRIMAGE (predictive *in silico* multiscale analytics to support cancer personalised diagnosis and prognosis, empowered by imaging biomarkers) is a funded Horizon 2020 project (RIA, topic SC1-DTH-07-2018) where a combination of these approaches ensures the best of both worlds.

**Fig. 1:**
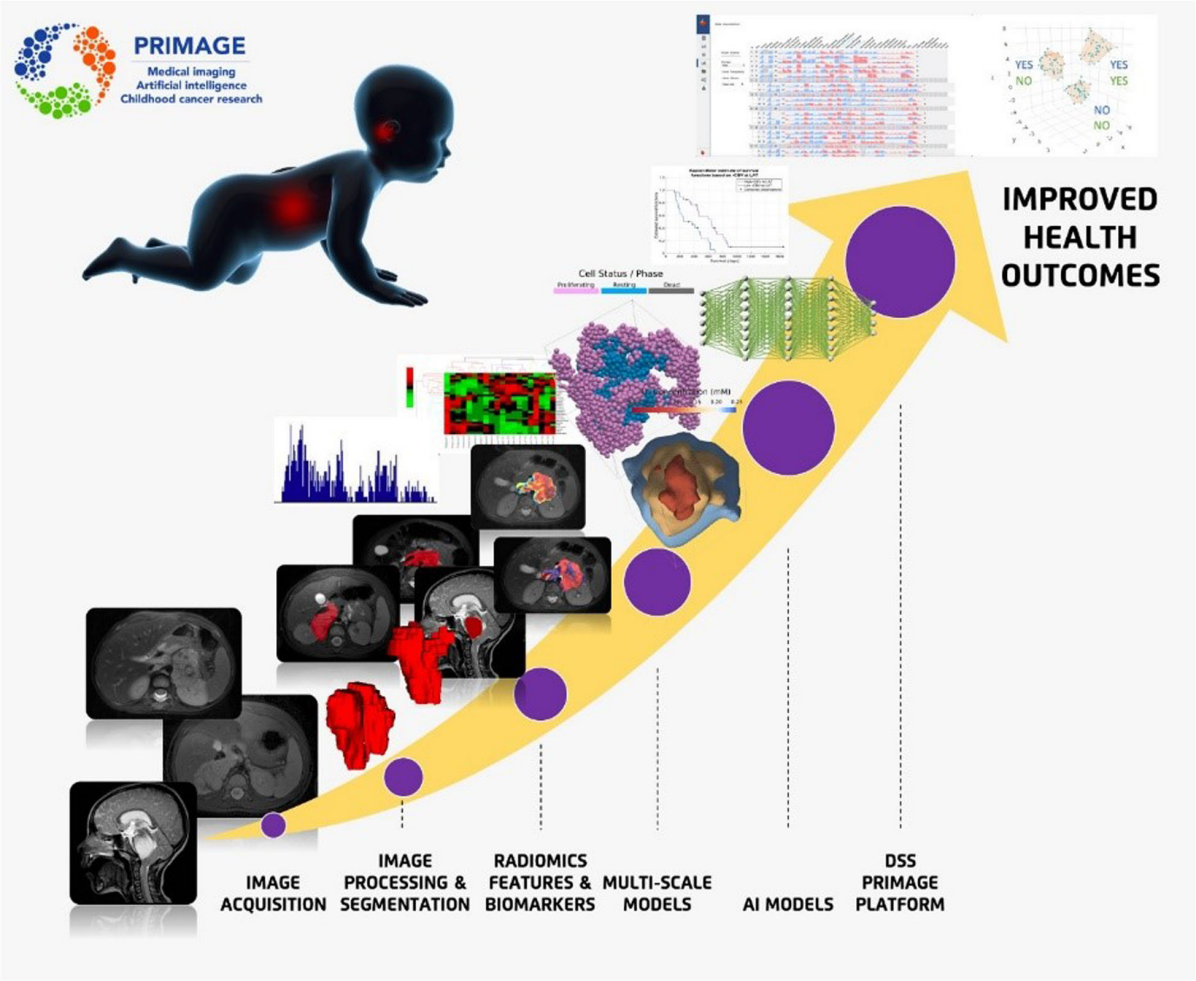
PRIMAGE technological development

The PRIMAGE project focuses on the further development of *in silico* tools for a more personalised clinical management of childhood cancer by targeting clinical endpoints (CEPs), considering the progression of the growth of the tumour post-diagnosis, but not including the initial oncogenic processes active during embryogenesis. The project will utilise novel high-performance computing (HPC) approaches to provide computationally efficient and large scale *in silico* models resulting in a decision support system (DSS) which will hopefully provide improved health outcomes (Fig. 1).

### Neuroblastoma and diffuse intrinsic pontine glioma (DIPG)

Data infrastructure, imaging biomarkers and models for *in silico* medicine research will be developed and validated in the context of neuroblastoma (NB) and diffuse intrinsic pontine glioma (DIPG).

NB is the most frequent solid cancer of the early childhood [13], and the diagnosis age has proven to be a crucial factor in its prognosis [14]. A number of risk factors have been identified and are already in use by the International Neuroblastoma Risk Group (INRG) [15, 16]. Major European groups involved in PRIMAGE have advanced standard of care treatments in low [17–19], intermediate [20, 21] and high-risk NB [22–24].

DIPG is the leading cause of brain tumour-related death in children [25]. Given the rarity of childhood tumours, international cooperative networks are essential to agglutinate relevant retrospective data and/or prospective cases for clinical trials, facilitating identification of effective tools for earlier diagnosis and potentially effective therapeutics.

The aim of the project is the development of an open hybrid cloud and HPC platform with later implementation and validation in non-interventional trials, which will support decision-making in the clinical management of malignant solid tumours. The PRIMAGE platform will implement the latest advancement of *in silico* computational image analysis and modelling which may be run on central processing unit (CPU) or general purpose graphics processing unit resources as needed.

The results are expected to have great impact not only on NB and DIPG but also on the management of other malignant solid tumours, since the proposed methodologies for data management, *in silico* models and visualisation tools will be available to be transferred to other cancer types.

**Fig. 2:**
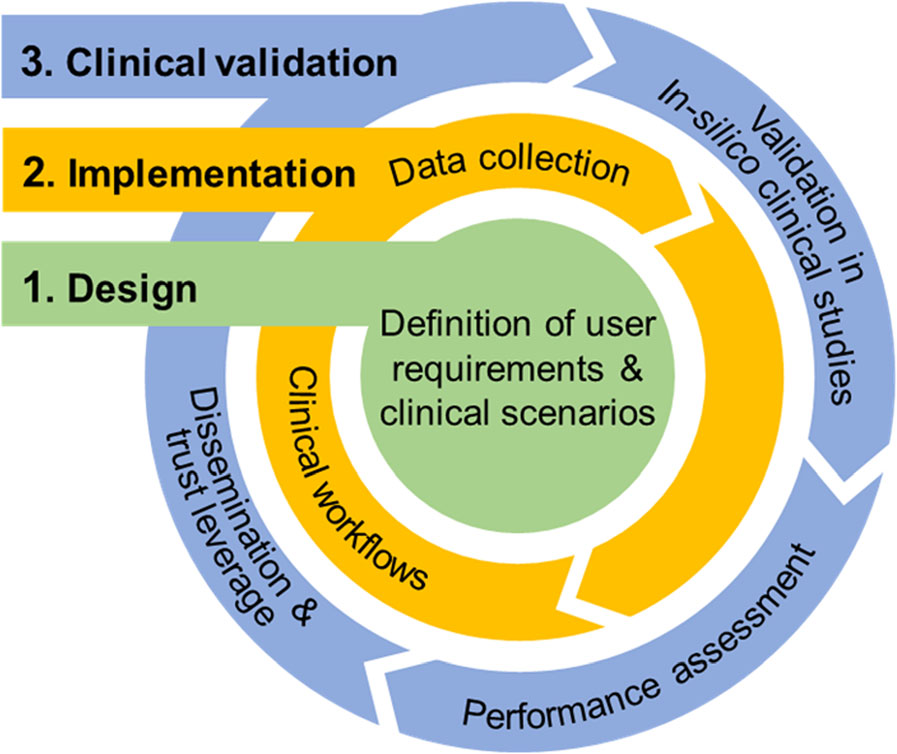
PRIMAGE approach

The development process of the PRIMAGE platform following a user-centric approach is summarised in Fig. 2.

## Methods

### The partnership

For the successful design and implementation of PRIMAGE, a very high level of interdisciplinarity consortium was required, with expertise ranging from HPC infrastructures to visual analytics [26] and multiscale simulation, bringing together public and private organisations across Europe to perform collaborative research and development, a key aspect of the PRIMAGE interdisciplinary approach. Partners from eight European countries were selected (hospitals, research and development centres, medical associations, private companies, and universities), constituting a pan-European ecosystem of knowledge, infrastructures, biobanks, and technologies in the area of oncology, *in silico*, and cloud computing/HPC healthcare-related computing. Following a recruitment process, 16 organisations were incorporated, all of them are well known and with remarkable expertise in their respective areas. The University and Polytechnic Hospital La Fe in Spain is the project coordinator institution.

On the clinical side, the PRIMAGE consortium has tried to reunite leading European clinical centres together with key opinion leaders specialised in NB and DIPG (as these cancers are the context of the application proposed for the validation works of PRIMAGE *in silico* tools during this project) (Table 1). The University and Polytechnic Hospital La Fe in Spain, the Children’s Cancer Research Institute in Austria, the University Hospital Cologne in Germany, and the Pisa University Hospital in Italy constitute the clinical team. All of them belong to the European Research Network in NB and DIPG, facilitating PRIMAGE access to their datasets for further secondary used studies, in addition to their own biobanks and databanks of existing DIPG and NB cases. Moreover, the European Society for Paediatric Oncology (SIOPE) joined PRIMAGE, leading its dissemination and communication activities.

**Table 1:** Clinical centres and networks data registries involved in data collection with an estimation of cases for neuroblastoma (NB) and diffuse intrinsic pontine glioma (DIPG)

Responsible entity	Characteristics
**Clinical partners for NB and DIPG** University and Polytechnic Hospital La Fe, Spain Children’s Cancer Research Institute, Austria University Hospital Cologne, Germany Pisa University Hospital, Italy	Target sample: approximately 900 cases with imaging, clinical, and molecular data. Data type: imaging (magnetic resonance, computed tomography, ^131^I-metaiodine-benzylguanidine scintigraphy and single-photon emission tomography, positron emission tomography/computed tomography), histology (if available), complete molecular biology studies according to SIOPE (blood, urine, and bone marrow, cerebrospinal fluid), genetic (next generation sequencing, fluorescence *in situ* hybridisation), and clinical data (patient profile, prescribed treatment, survival).
**Data on patients with NB** GPOH	Target sample: approximately 1,000 NB (high, low, and intermediate risk) patients participants in academia-promoted clinical trials. Data type: diagnosis and longitudinal data (clinical, follow-up, and biology data for all patients registered in GPOH database).
**Data on patients with DIPG** SIOPE registry	Target sample: approximately 700 DIPG patients from European Union countries, both inside and outside clinical trials. Data type: diagnostic and follow-up magnetic resonance scans linked to e-data transmittal form including demographics, medical history, and physical exam at time of diagnosis, results from radiological, results from pathological review (if available), treatment (including radiotherapy, chemotherapy, surgery and supportive), clinical data, and last known status of the patient.

*GPOH* German Society of Paediatric Oncology and Haematology, *SIOPE* European Society for Paediatric Oncology

The knowledge and expertise of universities, research centres and private companies are essential for successful development of PRIMAGE. Partners include Quantitative Imaging Biomarkers in Medicine (QUIBIM SME), Institute for Molecular Imaging Technologies and Mechanical Engineering Department at Valencia Polytechnical University, Chemotargets SME, and Matical Innovation in Spain; the Department of Computer Science at University of Konstanz in Germany; Medical Imaging Technologies (Medexpim) in France; the University of Sheffield in United Kingdom; Simulation, Modelling and Engineering software (Ansys group) in France; Akademia Gorniczo-Hutnicza Im (Cyfronet) in Poland; and the Department of Industrial Engineering at the University of Bologna in Italy.

The consortium partners ensure complementarity and bring the necessary combination of skill, knowledge, technology, and motivation, comprising a highly motivated team, fully committed to turning PRIMAGE into a case study in the use of existing datasets, service-oriented architectures and *in silico* technologies for better diagnosis and treatment of oncology diseases.

An advisory board consisting of a recognised group of experts in the fields of paediatric oncology, imaging biomarkers and related information and communication technologies, General Data Protection Regulation and industry representatives of manufacturers of drugs and picture archiving and communication systems has been designated to give general advice and guidance to the consortium.

### Platform architecture

The PRIMAGE *in silico* models to be developed require significant computational and data storage resources to process. Our intention is to deliver a bespoke information technology solution, combining large-scale HPC and versatile cloud computing resources for optimum efficiency and reliability.

**Fig. 3:**
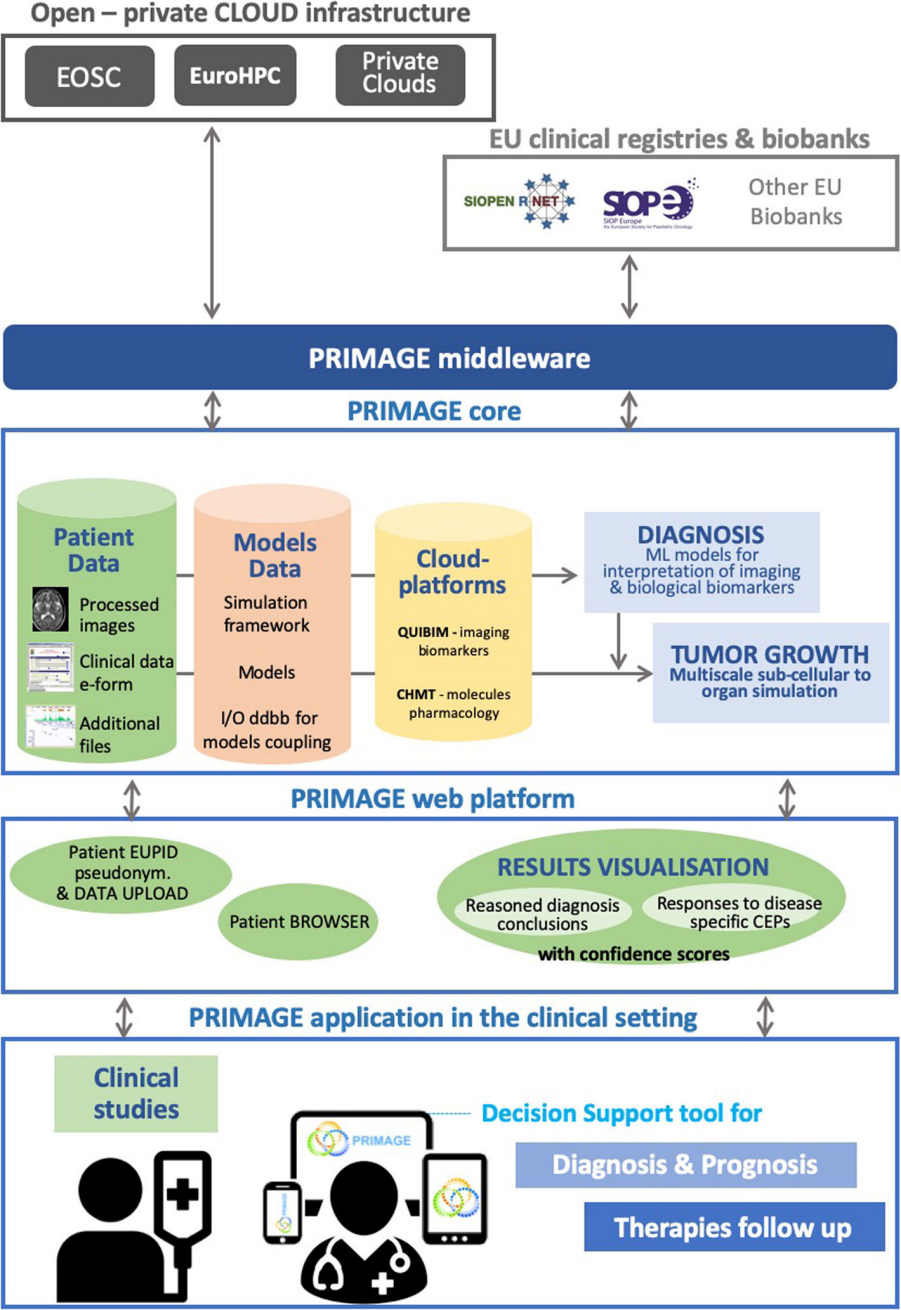
PRIMAGE platform concept diagram

The infrastructure will be ultimately based on a DSS which will be designed for cancer management with advanced functionality and usability under a user-centric approach, guided by the clinical partners. A diagram of a high-level architecture can be appreciated in Fig. 3. This DSS will make use of the following:

- Large-scale processing on HPC resources (CPU or general purpose graphics processing unit), overlaid by a convenient representational state transfer-based process controller called Rimrock [27] and data access suite called Polish Grid Infrastructure (PL-grid) Data [28].
- Hybrid cloud resources, composed of both private and public cloud sites (based on the Europen open science cloud, EOSC, services), which will host PRIMAGE data repositories. Computational tasks can be deployed and coherently managed by a single access tool, such as the Atmosphere platform (project number 777154, European Commission) [29].
- An integration middleware, consisting of a set of protocols and interfaces between HPC/storage (models), private and public cloud computing/storage (repositories, sandboxed processing), and external data sources (anonymised clinical and biobanking data). The middleware will be put in place to achieve an adequate level of solution coherency.
- Upper layer service exposing the features of the underlying infrastructure to researchers as a convenient graphical user interface, to manage definition, execution, and comparison of results of computationally intensive modelling pipelines. The tool will feature security management and application programming interface for programmatic access, and it will be based on the model execution environment (MEE) [30], which has been developed and successfully deployed in the EurValve project [31–33].

### Data repositories

Stored clinical data (imaging, clinical, pathology and molecular) from 2002 onwards will be collected by the clinical centres and the paediatric oncological associations involved in PRIMAGE project. Clinical, molecular and imaging data in PRIMAGE will be undertaken under the strictest administrative and contractual procedures to ensure legal and ethical compliance under the General Data Protection Regulation in Europe. All cases included within the data repository, both for model development and platform validation, will have to be approved by the Ethics Committees of their respective centres. Even more, the observational *in silico* trial design for the internal platform validation, including new data from 2020 to 2022, will be constructed in parallel to standard clinical practice after signed consent is obtained.

Relevant data will be used following extraction, anonymisation and curation for computational simulation developments and testing the integrated PRIMAGE DSS platform. The datasets used for the training and the development of the different *in silico* models will differ from the datasets used for the later validation of the integrated platform. Therefore, the use of clinical data (in the big data domain) for model training and model testing will be done in two phases:

- Phase 1: compilation of clinical, molecular and imaging data for PRIMAGE training, knowledge extraction, and multiscale testing of the *in silico* models for tumour growth, advanced visualisation solutions, identification and analysis of imaging biomarkers and training of predictive models for CEPs.
- Phase 2: extensive *in silico* testing of the integrated PRIMAGE DSS platform will be undertaken using cases from the same retrospective dataset, split by cross validation from the curated initial clinical dataset to ensure that these specific datasets are not used in the training phase.

Big data techniques will be applied to generate new knowledge from the advanced *in silico* tools. The use of retrospective data for training models, testing and validation will require extensive curation and quality control procedures. Automated tools will be implemented in this project to streamline the processes of extracting, mapping data, controlling the quality and homogenisation, translation, and completion of data feeds. Allocation of significant human resources is also foreseen, as human intervention is essential to achieve excellence in the training data sets.

Clinical data used in the international staging and stratifying criteria for NB and DIPG, including age, disease extension, image defined risk factors, histological type, grade of tumour differentiation, genetic, and molecular features will be used at the models construction phase. Imaging biomarkers will be extracted and validated for their use in cancer management, in combination with already available biological biomarker panels. PRIMAGE is defined as an observational *in silico* study that will be validated also *in silico* in data recorded from new clinical observations. Image radiomics and dynamic parameters will be obtained from standard of care real-world ultrasound, computed tomography, magnetic resonance, positron emission tomography, and ^123^I-metaiodobenzylguanidine scintigraphy or single-photon emission computed tomography images.

Experimental and computational methodologies will be used for the identification and validation of novel imaging biomarkers and the development of shared instruments for knowledge extraction from imaging biomarkers, clearly focused on improving disease diagnosis and follow-up. The development of a biomarker involves defining its relationship with the objective reality (structural, physiological, biological, or molecular), monitoring its technical validity and the relationship with the final CEPs. The path to handcrafted biomarker development, expansion and subsequent implementation is based on available guidelines and recommendations [34]. Main outcomes to be predicted relate to tumour phenotyping, treatment allocation, response to treatment and children survival.

Machine learning and image processing deep learning algorithms extract pattern information from the images and link outcome results to known ground-truth diagnosis. For this task, QUIBIM, a start-up company working in the field of artificial intelligence and imaging biomarkers applied to radiology data, will provide the methodologies for identification and validation of imaging biomarkers and related algorithms for the automatic analysis of images towards their validation in clinical trials through a PRIMAGE-specific customisation of QUIBIM precision platform (SME Instrument Phase 2, project number 778064). The computational equation-based handcrafted methods will be used first to have a ground truth for the training of machine learning algorithms. On a furthermore advanced stage, at the end of the project, it is envisioned that deep learning tools will directly provide clinical estimations from the source images, clinical and molecular data information.

### *In silico* scale models

PRIMAGE’s proposed *in silico* model spans three scales:

- *▪ The tumour scale model* describes the evolution of volume and cellularity in the primary tumour, as well as its biomechanical interactions with the surrounding tissues, and the diffusion of nutrients and metabolites to and from the closest blood vessels. These quantities are described as spatial fields, whose temporal evolution is governed by a system of partial differential equations. These equations are discretised and then integrated over a period of time using a finite element method. Reduced order techniques will be applied on tumour parameters (variability of tumour size, material properties and treatment type) to enable real-time simulation at the tumour scale.
- *▪ The tissue scale model* requires two complementary methodologies—firstly, a continuous, partial differential equation-based model that includes the chemical, biological and biomechanical interactions of the NB/DIPG cells with each other and with their extracellular matrix and vasculature. Model outputs will be the population sizes of different cell types and their evolution over time. In parallel to this, we will develop an agent-based model [35], wherein each cell is represented as an individual within a region of interest, explicitly capturing cellular behaviours including cell cycle progression, cell cycle arrest, cell death, production and degradation of the extracellular matrix, physical intercellular interactions, and cell-microenvironment interactions. This model may be used independently to explore hypotheses about specific regions of the tumour at a high resolution, or be fully integrated with other PRIMAGE models using state-of-the-art optimisation and acceleration techniques [36] and multiscale modelling approaches, as described below.
- *▪ The cell scale model* describes the evolution of chemical and biological properties over time inside a single NB/DIPG cell, when it is exposed to different environmental conditions including various treatments.

### Computational strategy

This project proposes a dataflow strategy to enable the proposed multiscale model to be executed using available HPC resources, in an effective and robust manner, considering that the proposed tumour model will need to be coupled to hundreds of thousands of tissue models, each coupled to also hundreds of thousands cell models.

In the proposed dataflow strategy, each single-scale model is described as a black box that takes as input an array of input sets and produces as output an array of output sets. Bi-directional resampling modules are proposed between the database that contains the input sets as computed, and those as required by the next model. Thus, resampling modules are used on value sets that are computed at lower scales and homogenised at upper scales, and on value sets that are computed at upper scales and then particularised at lower scales, at each scale transition.

The proposed workflow for the implementation of the described dataflow approach is:

- ▪ Implementation of the software infrastructure to manage the multiple databases. This infrastructure includes frameworks for coupling of the models at different scales via resampling functions, and repositories for the three single-scale models as they develop.
- ▪ Models execution without resampling. At this stage, no resampling will be provided as the software developments are focused on delivery and validation of the dataflow architecture to execute the multiscale model under suboptimal conditions, as efficiently as possible (using acceleration techniques).
- ▪ Models execution with resampling. Tissue-to-organ and cell-to-tissue modules will be incorporated in the dataflow, using progressively sophisticated multidimensional sampling techniques.
- ▪ Testing alternative approaches for enhancing computational efficiency. The use of surrogate modelling methods such as Gaussian processes will be explored as an alternative of resampling techniques. Although operating under completely different principles, they can be used to produce same result.

Even more, deep learning solutions will be developed, tested and validated for image segmentation (convolutional neural network architectures), radiomics analysis (support vector machine, linear discriminant, quadratic discriminant, decision trees, logistic regression, nearest neighbour, deep neural networks, and their combination in ensemble models) and CEPs (*e.g.*, tumour subtypes, patient’s prognosis), to estimate most accurate lesion diagnosis, treatment prediction, and patient prognosis. Models will be adapted to each application context (NB, DIPG) in order to more specifically address the clinical problems.

### Performance platform validation

The proposed *in silico* tools will help tackle the relevant CEPs on the clinical target applications. The following CEPs have been prioritised by the Consortium clinicians as highly relevant to these diseases and suitable for being supported by *in silico* models, contributing to enhance reliability of current diagnosis and prognosis procedures:

- Prognosis of NB spontaneous regression capacity for low and intermediary risk patients;
- Identification of high-risk NB patients with imminent risk of relapse (50% currently);
- Identification of NB high-risk patients that will not respond to induction chemotherapy (30% currently);
- Identification of DIPG patients who will respond to treatment (10% currently);
- Estimation of the expected survival period for DIPG responder patients.

A functional version of the PRIMAGE platform will be extensively evaluated in order to assess the platform’s performance, thus its capacity to guide clinical decisions in a precise, reliable, and relevant manner. The datasets used for the validations includes imaging data, clinical data and genetics and other molecular data (Table 2).

**Table 2:** Datasets used for testing of the PRIMAGE platform

**Imaging data**	Imaging data represents the highest challenge in terms of storage and processing. In PRIMAGE data repositories, for each patient, imaging data is linkable to their available pseudonymised biological, pathological, and genetics. The use of common metadata frameworks and image analysis techniques for automated data annotation for each image is proposed to generate common repositories
**Genetics and other molecular data**	This project uses existing knowledge on biological biomarkers (currently on clinical use or at advanced clinical validation stage). This type of data is used in combination with imaging and clinical data, facilitating multidisciplinary big data analytics.
**Clinical data**	Use of natural language processing tools for automated extraction of relevant pathological data, including data on patient response to specific treatment will be extracted from the electronic health record. Data will then be structured, curated, and stored.

The *internal validation* of PRIMAGE platform will be performed using datasets provided by the clinical centres and organisations involved in PRIMAGE project which never were used for the models and platform development.

In addition, in order to guarantee its correct performance under general conditions when using any datasets from the real world, PRIMAGE platform will be *externally validated* using data from clinical centres out of PRIMAGE environment. To get access to this data, other hospitals not involved in the project as partners have already been invited to participate by providing NB and DIPG cases to the PRIMAGE platform as independent collaborative international centres.

Table 3 describes the methodologies that will be used in the evaluation of PRIMAGE platform performance. During the evaluations, it will be also assessed other metrics such as security, reproducibility, interoperability and usability amongst others (Table 4).

**Table 3:** PRIMAGE platform testing methodologies and performance metrics

	Main testing methodologies	Main performance metric
Cloud infrastructure	Definition of unitary and integration tests based on the application requirements, monitoring along time, design-time vulnerability analysis.	Performance (deployment and reconfiguration overheads, performance penalties, scalability), reliability (correct results with respect references), reproducibility (predictability of performance and automation), robustness (reliability along time and under different stress conditions), security (identification of vulnerabilities and isolation), privacy (privacy risk estimation).
High-performance computing infrastructure	Continuous monitoring of infrastructure. Alerts for administrators in case of malfunctions or failures.	VM start-up time. Resource consumption. Number of concurrently running computational tasks. Availability. Measured through monitoring statistics, experiments and benchmarks.
Data repositories	Testing on MR, ^131^I-MBIG imaging, CT, PET/CT data, from retrospective studies of neuroblastoma and diffuse intrinsic pontine glioma patients	Correlation between clusters of imaging biomarkers. Correlation between radiomic signatures and genomic profiles, and/or circulating tumour biomarkers from liquid biopsy (circulating tumour cells, tumour nucleic acids, etc.)
Imaging biomarkers	Testing on images (MR, ^131^I-MBIG imaging, CT, PET/CT) from retrospective data of neuroblastoma and diffuse intrinsic pontine patients	Precision, accuracy and clinical relationship measured in terms of quantified limit of detection and limit of quantification, reproducibility, sensitivity/specificity, coefficient of variation, correlation to diagnosis/prognosis of a specific disease
Multiscale modelling framework	Qualitative and quantitative comparison of numerical predictions with retrospective data	Quantitative correlation of the shape and size of tumour between image-based data and computer-based results. Qualitative correlation of vascular level and extracellular matrix properties in the tumour surroundings.

*CT* Computed tomography, *MR* Magnetic resonance, *MBIG* Metaiodobenzylguanidine, *PET* Positron emission tomography

**Table 4:** Metrics assessed in PRIMAGE platform

Metrics to be assessed	Methodology
Security/privacy	Provision of authentication and authorisation and analysis of vulnerabilities from public databases. Assessment of the platform’s robustness to preserve data integrity according to GDPR, evaluating the privacy risk (*e.g.*, as the capability of a model to infer information previously anonymised), and managing the fine-grain consent as GDPR requires.
Correctness/reliability	Assess the correctness of the predictive results for the established clinical end points using testing datasets from clinical data repositories for over 2000 neuroblastoma patients and over 500 diffuse intrinsic pontine glioma patients with complete diagnosis and follow-up data, including treatment and outcomes.
Sensitiveness to incomplete data	Assess dependence of correctness of the predictive results with the completeness of the diagnosis datasets, to establish how new biomarkers modify minimum datasets required for correct diagnosis/prognosis.
Reproducibility	Statistical assessments: dispersion in the results obtained for a subgroup of patients with a common clinical diagnosis, belonging to different hospitals where the diagnosis studies were undertaken
Interoperability	Assessment of failures in the integration with hospital picture archive and communication system and electronic health record systems, as well as to on-premise and public cloud services
Malfunction	Occurrence of any fatigue of integrity or potential to induce to use errors
Relevance	Interviews to assess users’ own judgement of helpfulness of the platform to guide them beyond obvious decisions for a given data available set
Added value	Statistical assessment of occurrence of correct predictions for clinical end points that current diagnosis/prognosis standard protocols could not predict correctly
Usability/user friendliness	Observation of use patterns, users’ eye tracking, interviews to users

*DIPG* Diffuse intrinsic pontine glioma, *GDPR* General Data Protection Regulation

## Expected results

The state of the art for production-quality hybrid computational cloud and HPC for *in silico* processing of clinical cases is currently represented by projects such as EurValve [30, 31, 37] and GoSmart [38, 39], having achieved significant progress in developing integrated, comprehensive frameworks. EurValve has also come up with an integrated cloud/HPC computing solution to back up its MEE, dedicated for simulations of valvular heart conditions. This environment is now used in EurValve to perform clinical validation of the resulting DSS, which is a preliminary step towards development of an integrated, non-distributed clinical DSS.

The significant novelty of PRIMAGE with regard to this state of the art is to bring the cloud and HPC computing solutions, already successfully utilised for development and validation of *in silico* models, considerably closer to clinical use. The project will carefully evaluate available strategies, and it will deploy the selected solution to remotely provision the computationally intensive elements of the proposed clinical DSS, thus enabling the advantages of in-cloud computation for today’s DSSs. The PRIMAGE approach combines the training and validation of models for medical imaging biomarkers and tumour growth simulation on open scientific cloud infrastructures, which constitutes the most computing intensive part, with the use of those models for personalised diagnosis, prognosis and optimisation of treatment, within hospital boundaries. The construction of *in silico* clinical trials will allow decision-making from causal inference from observational databases emulating a pragmatic target trial if methodological pitfalls are avoided [2].

This project will bring major advancements in the validation of novel imaging biomarkers; it will create advanced computational models for tumour growth simulation, given response to specific CEPs. A very limited number of imaging biomarkers have been used in routine clinical practice to guide clinical decisions [7–9]. Therefore, PRIMAGE predictive models will enable unprecedented effectiveness in the translation from clinical Big Data to personalised predictors for malignant solid tumours, particularly NB and DIPG, by incorporating these assets for Big Data to usable clinical knowledge translation. The PRIMAGE diagnosis guiding tool uses quantification of imaging findings to improve sensitivity, specificity, accuracy and reproducibility of diagnostic and therapeutic decisions derived from image features used in combination with clinically validated biological biomarkers and cross-cohort machine learning from European repositories for NB and DIPG. We aim to impact current clinical guidelines [40].

PRIMAGE is designed to have a major impact on improving the disease management of malignant solid tumours. Upon successful validation in NB and DIPG, evidencing how *in silico* tools can assist clinicians to make improved informed decisions, PRIMAGE platform’s architecture, repository infrastructure, simulation frameworks, and web-based user interfaces will have the potential to be adapted and completed for use in the management of other types of cancer.

We do not foresee any intellectual property nor commercial issues during the project: the intellectual property background of each partner has been defined and declared already, as well as the general framework on the future exploitation and ownership of the project’s results. At the end of the project, there will be pieces of software, computational models and other intellectual property assets that will be owned by the partner(s) that developed them. Exploitation and/or access rights on these developments may be given to the rest of partners under favourable conditions. This shall be set in specific commercial agreements between the owner(s) and the licensee(s) after the end of the project. Finally, regarding the access to the PRIMAGE platform as a whole, the aim of the project is to give open access to the research community. Means for the sustainability and continuity over time of the PRIMAGE platform will be assessed also during the project.

## Conclusion

At the end of the project, the developed open cloud-based platform will support phenotyping (diagnosis), treatment stratification (prediction) and patient-specific CEPs determination (prognosis), based on the use of imaging biomarkers, tumour growth simulation, advanced visualisation of confidence scores, and machine learning approaches. The decision support prototype will be constructed and validated on NB and DIPG cancers. The results will be available for the scientific community and ready for transfer learning to other malignant solid tumours. Data infrastructures, imaging biomarkers, and predictive models for *in silico* medicine research will be validated during this project. Given the rarity of these tumours, international cooperative networking is essential to agglutinate relevant *in silico* data from clinical trials and large real-world data repositories, facilitating identification of effective clinical tools.

## Data Availability

Project website (https://www.primageproject.eu/)

## Abbreviations

API: Application programming interface
CEPs: Clinical endpoints
CPU: Central processing unit
DIPG: Diffuse intrinsic pontine glioma
DSS: Decision support system
HPC: High-performance computing
INRG: International neuroblastoma risk group
MEE: Model execution environment
NB: Neuroblastoma
PRIMAGE: Predictive in-silico multiscale analytics to support cancer personalised diagnosis and prognosis, empowered by imaging biomarkers
QUIBIM: Quantitative Imaging Biomarkers in Medicine
SIOPE: European Society for Paediatric Oncology
